# Sociocultural Perspectives of Abuse of Older Adults within Family Caregiving Settings in Nigeria

**DOI:** 10.1093/geroni/igaf122.385

**Published:** 2025-12-31

**Authors:** Tochukwu Okolie, Patricia Agbawodikeizu, Prince Ekoh, Ngozi Chukwu, Emmanuel Ugwu, Samuel Onuh, Obinna Odo, Kingsley Udeh

**Affiliations:** University of Connecticut, Mansfield Center, Connecticut, United States; University of Nigeria Nsukka, Nsukka, Enugu, Nigeria; University of Nigeria Nsukka, Nsukka, Enugu, Nigeria; University of Nigeria Nsukka, Nsukka, Enugu, Nigeria; University of Nigeria Nsukka, Nsukka, Enugu, Nigeria; University of Nigeria Nsukka, Nsukka, Enugu, Nigeria; Miami University, Oxford, Ohio, United States; Miami University, Oxford, Ohio, United States

## Abstract

In Nigeria, family members remain at the core of providing care to older adults. However, these intrinsic and culturally accepted responsibilities are occasionally contrasted/undermined by inadvertent abuse of the older person within family caregiving settings. Notably, what is considered abusive or not in a caregiving situation is usually culturally defined. Therefore, this study explored the perspectives of older adults about caregiving behaviors that they consider to be abusive. Data were obtained using semi-structured interviews with 16 older adults age 60 years and above in a rural community in Awgu Local Government Area (LGA), Enugu state, Nigeria. The data were analyzed thematically with the aid of NVivo12 software. Five themes emerged as caregiving behaviors that were considered abusive (i) neglect of caregiving responsibilities (ii) forced feeding and/or medication (iii) nonconsensual use of older adults’ money/assets (iv) restriction of older adults’ movements (v) invasion of privacy. Majority of the participants in the study verbalized a detest for these behaviors by family members, while others added that sometimes those were normal and expected caregiving behaviors. The caregiving behaviors identified by participants in this study are usually not within the typically reported types of abuse of older adults in Nigeria. However, they may constitute detrimental consequences to the health and quality of life of older adults in Nigeria. Hence, the study recommends research into these specific caregiving behaviors to understand how and if they impact older adults’ overall quality of life in any way.

